# Analysis of *Gga* Null Mice Demonstrates a Non-Redundant Role for Mammalian GGA2 during Development

**DOI:** 10.1371/journal.pone.0030184

**Published:** 2012-01-26

**Authors:** Jennifer Govero, Balraj Doray, Hongdong Bai, Stuart Kornfeld

**Affiliations:** Department of Internal Medicine, Washington University School of Medicine, St. Louis, Missouri, United States of America; Institut Curie, France

## Abstract

Numerous studies using cultured mammalian cells have shown that the three GGAs (Golgi-localized, gamma-ear containing, ADP-ribosylation factor- binding proteins) function in the transport of cargo proteins between the *trans*- Golgi network and endosomes. However, the *in vivo* role(s) of these adaptor proteins and their possible functional redundancy has not been analyzed. In this study, the genes encoding GGAs1-3 were disrupted in mice by insertional mutagenesis. Loss of GGA1 or GGA3 alone was well tolerated whereas the absence of GGA2 resulted in embryonic or neonatal lethality, depending on the genetic background of the mice. Thus, GGA2 mediates a vital function that cannot be compensated for by GGA1and/or GGA3. The combined loss of GGA1 and GGA3 also resulted in a high incidence of neonatal mortality but in this case the expression level of GGA2 may be inadequate to compensate for the loss of the other two GGAs. We conclude that the three mammalian GGAs are essential proteins that are not fully redundant.

## Introduction

The GGAs (Golgi-localized, gamma-ear containing, ADP-ribosylation factor- binding proteins) are a family of monomeric clathrin adaptor proteins that facilitate trafficking of cargo proteins from the *trans*-Golgi network (TGN) to endosomes [1]. Mammals contain three GGAs, termed GGA1, GGA2, and GGA3. These proteins have identical domain organizations with an N-terminal VHS (VPS-27, Hrs, and STAM) domain followed by a GAT (GGA and Tom1) domain, a connecting hinge segment and a C-terminal GAE domain that is homologous to the ear domain of γ-adaptin [2]. The VHS and GAT domains of the three mammalian GGAs are highly conserved with 60–75% identity [3].

The GGAs are recruited from the cytosol onto the TGN via binding of the GAT domain to ARF•GTP and PI4P [3]–[5]. The VHS domain then binds acidic-cluster dileucine (AC-LL) sorting motifs present in the cytoplasmic tails of cargo molecules and facilitates the incorporation of these molecules into forming clathrin-coated carriers which deliver them to endosomes [2]. Cargo molecules known to interact with the GGAs include the cation-independent and cation-dependent mannose 6-phosphate receptors, sortilin, sortilin-related receptor (SorLA/LR11), β-secretase (BACE1), low-density lipoprotein receptor-related proteins 3, 9 and 12, stabilin-1, consortin, and chloride channel 7 [6]–[16]. In addition to functioning at the TGN, GGA3 has also been implicated in the sorting of ubiquitinated cargo at the endosomes [17], [18].

A critical question is whether the three mammalian GGAs have distinct roles or are functionally redundant, as is the case with the two yeast GGAs [19], [20]. Since none of the cargo proteins known to interact with the mammalian GGAs have been found to bind exclusively to one particular GGA, it has generally been assumed that these GGAs are likely redundant. However, the GGAs do exhibit some differences in their binding to AC-LL motifs of the various cargo proteins [8], [10], [16], [21], [22]. In addition, human GGA1 and GGA3 but not GGA2 are phosphorylated *in vivo* and subject to autoinhibition mediated by binding of internal AC-LL motifs present in the hinge to the ligand binding site on the VHS domain [23], [24]. The GAT domains of human GGA1 and GGA3 but not GGA2 bind ubiquitin and ubiquitinated proteins [25], [26]. Finally, GGA2 has a shorter half-life than GGA1 and GGA3 [27].

It is also of note that the amino acid differences between the GGAs are highly conserved among species. Thus, mouse GGA1 is more similar to human GGA1 (91.1% identity) than human GGA1 is to human GGA2 (47.7% identity), etc, as determined by the SIM Alignment Tool of the ExPASy Proteomics Server (Swiss Institute of Bioinformatics). This is consistent with each GGA having a function not fully shared with the other GGAs. However, attempts to establish specific roles for the individual GGAs have been inconclusive. There are a number of reports where one or more of the GGAs were knocked down in tissue culture cells using RNAi and the impact on the trafficking of cargo molecules was followed [28]–[32]. None of these studies established a distinct role for any of the three GGAs. Further, since all of the knock-down studies were performed with cells in tissue culture, the *in vivo* role of the GGAs in mammals has yet to be evaluated.

We now report the consequence of disrupting the genes encoding GGA1, GGA2 and GGA3 in mice using insertional mutagenesis. The major finding is that loss of GGA2 is lethal whereas loss of GGA1 or GGA3 alone is well-tolerated. This establishes that GGA2 mediates a function that cannot be compensated by GGA1 and GGA3.

## Results

### Generation of *Gga1*, *Gga3* and *Gga1/3* null mice

**Figure 1:**
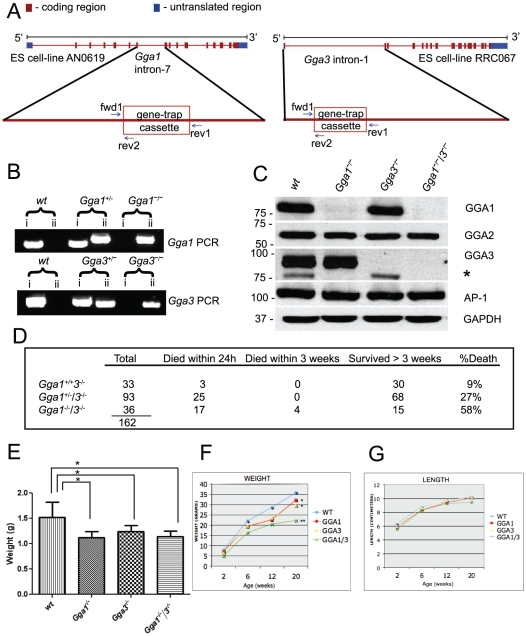
Analysis of *Gga1*, *Gga3* and *Gga1/3* null mice. A) The position of the gene-trap within intron-7 of the mouse *Gga1* gene is shown along with the position of the gene-trap within intron-1 of the mouse *Gga3* gene. As a consequence of these gene-traps, normal mRNA is disrupted by the splice acceptor site of the engrailed-2 leader sequence fusing with either codon-203 (encoding E203) of the mouse *Gga1* gene or codon-14 (encoding S14) of the mouse *Gga3* gene. *Gga1* and *Gga3* PCR primer sets fwd1/rev1 and fwd1/rev2 (Figure S1A) were used to distinguish between wt and null genotypes, respectively. B) PCR of genomic DNA was performed using *Gga1* specific primers, (*Gga1* PCR, reaction i) *Gga3* specific primers (*Gga3* PCR, reaction i), and a *Gga1* specific primer or *Gga3* specific primer in combination with a gene trap specific primer (reactions ii). PCR band in reactions (i) indicates wt genotype, while PCR band in reactions (ii) indicates null genotype. C) Western blot analysis of mouse brain lysates probed for GGA1, GGA2, GGA3, AP-1 and GAPDH (loading control). * denotes a GGA1- cross reacting band (see Figure S4). D) *Gga1^+/−^/Gga3^−/−^*×*Gga1^+/−^/Gga3^−/−^* matings were set up and the resulting mice were genotyped by PCR as described in panels A and B. E) Birthweights were obtained on day 1 and plotted according to genotype (*wt*, n = 10; *Gga1* ^−/−^, n = 23; *Gga3* ^−/−^, n = 20; *Gga1* ^−/−^/*3* ^−/−^, n = 20). * represents p = <0.001; as determined by Student's t-test. F and G) Weights and nose-rump lengths (NRL) of wt (blue), *Gga1^−/−^* (red), *Gga3^−/−^* (yellow) and *Gga1^−/−^/3^−/−^* (green) mice were obtained at the times indicated and plotted versus age in weeks. * represents p<0.05, ** represents p<0.001; as determined by Student's t-test.

**Figure 2:**
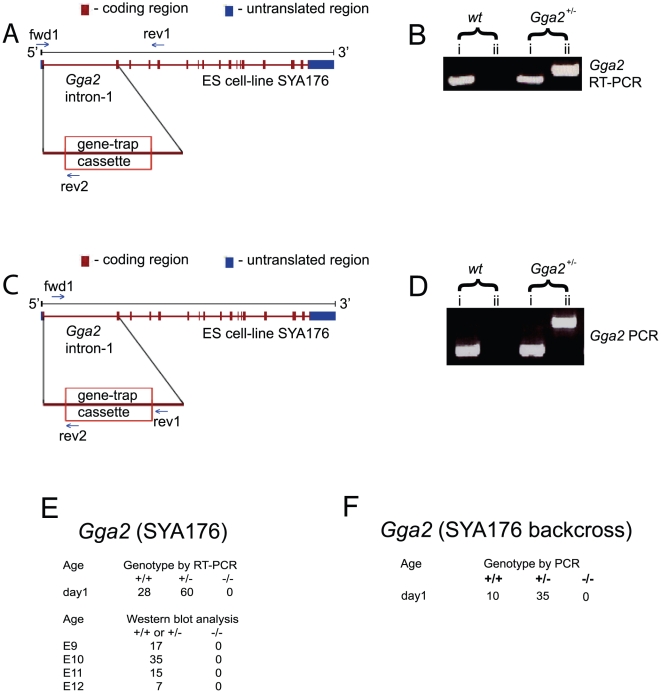
Analysis of *Gga2* null mice (SYA176 strain). A) Insertion of the gene-trap cassette within intron-1 of the mouse *Gga2* (SYA176) gene results in normal GGA2 mRNA being disrupted by the splice acceptor site of the engrailed-2 leader sequence fusing with codon-33 (encoding L33) of the mouse *Gga2* gene. The progeny of *Gga*2^+/−^ inter-crosses were genotyped by subjecting cDNA to RT-PCR with *Gga2* primer sets fwd1/rev1 (spanning exons-1 through 5) and fwd1/rev2 (spanning exon-1 and the β-galactosidase exon) (Figure S1A), which differentiated between the wt and null genotypes, respectively. B) RT-PCR of cDNA prepared from white blood cells or brain tissue was performed using *Gga2* specific primers (reactions i) or a *Gga2* specific primer in combination with a gene trap specific primer (reactions ii). PCR band in reactions (i) indicates wt genotype, while PCR band in reactions (ii) indicates null genotype. C) Determination of the insertion site of the gene-trap within intron-1 of the mouse *Gga2* gene (SYA176) allowed for the design of *Gga2* primer sets fwd1/rev1 and fwd1/rev2 (Figure S1A), which clearly distinguished between wt and null genotypes, respectively, at the gene level. D) PCR of genomic DNA was performed using *Gga2* specific primers (reactions i) or a *Gga2* specific primer in combination with a gene trap specific primer (reactions ii). PCR band in reactions (i) indicates wt genotype, while PCR band in reactions (ii) indicates null genotype. E) The genotypes of the *Gga2^+/−^* crosses were determined either by RT-PCR (day1 pups) or Western blots (embryos E9, E10, E11 and E12). No homozygous null were obtained out of 162 newborns or embryos that were analyzed. F) PCR was performed using genomic DNA from newborn pups of SYA176 *Gga2^+/−^* crosses in the C57BL/6J background. No homozygous nulls were obtained out of 45 pups analyzed.

**Figure 3:**
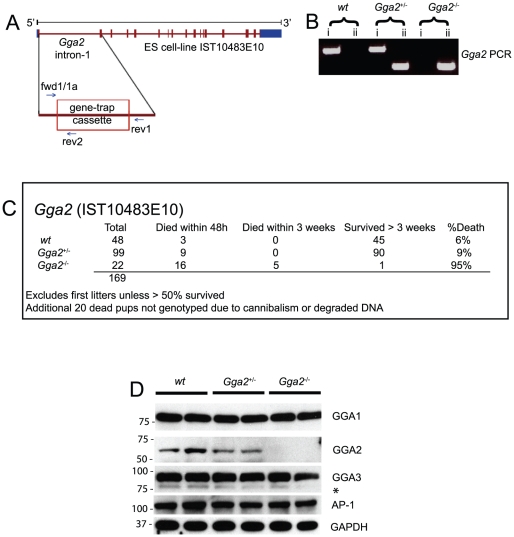
Analysis of *Gga2* null mice (TIGM strain). A) The position of the gene-trap within intron-1 of the mouse *Gga2* gene (cell line, IST10483E10) is shown. As a consequence of this gene-trap, normal messenger RNA (mRNA) is disrupted by the splice acceptor site of the engrailed-2 leader sequence fusing with codon-33 (encoding L33) of the mouse *Gga2* gene. *Gga2* primer sets fwd1/rev1 and fwd1a/rev2 (Figure S1A) were used to clearly distinguish between wt and null genotypes, respectively. B) PCR of genomic DNA was performed using *Gga2* specific primers (reactions i) or a *Gga2* specific primer in combination with a gene trap specific primer (reactions ii). PCR band in reactions (i) indicates wt genotype, while PCR band in reactions (ii) indicates null genotype. C) PCR was performed using genomic DNA from newborn pups of TIGM *Gga2^+/−^* crosses. 169 pups were analyzed and homozygous insertion of the gene-trap into the *Gga2* gene was determined to be 95% lethal by 3 weeks of age. D) Western blot analysis of TIGM *Gga2* mouse brain lysates probed for GGA1, GGA2, GGA3, AP-1 and GAPDH. * denotes a GGA1- cross reacting band (see Figure S4).

To elucidate the role of the GGAs *in vivo*, mice deficient in each of the GGAs were generated via insertion of a gene-trap cassette (Figure 1A; Figure 2A and C; Figure 3A). Mouse embryonic-stem (ES) cell-line ID AN0619 containing a gene-trap cassette inserted within intron-7 of the mouse *Gga1* gene, and ES cell-line ID RRC067 containing a gene-trap cassette inserted within intron-1 of the mouse *Gga3* gene were used to obtain founder heterozygous (het) mice in a C57BL/6J and 129/Ola mixed genetic background (Figure 1A). Progeny of the het matings were analyzed by PCR (Figure 1B), and revealed a genotype distribution of the expected Mendelian ratios for *Gga1* and *Gga3* offspring (not shown). A two-step mating scheme starting with *Gga1* het/*Gga3* null mice was used to obtain the *Gga1/Gga3* double null mice (*Gga1/3* null), which were born in accordance with the expected Mendelian ratios (Figure 1D).

Immunoblot analysis was performed on mouse brain lysates to confirm the effect of the gene targeting on protein expression. As illustrated in Figure 1C, GGA1 was either undetectable or occasionally showed trace amounts (less than 1% of wt) in the *Gga1* and *Gga1*/3 null brain lysates, which we attribute to a low-level of read-through transcription and alternate splicing around the gene-trap cassette. The GGA1-β-galactosidase (β-gal) fusion protein made in the *Gga1* and *Gga1*/3 null strains encodes the first 203 amino acids corresponding to exons-1 through 7 (Figure 1A). We do not believe this fusion protein could function as a dominant negative since it is lacking most of the GAT domain, which is required for the inhibitory effect of the dominant negative construct [4]. GGA3 was undetectable in the *Gga3* and *Gga1*/3 null brain lysates (Figure 1C). The GGA3-β gal fusion protein encodes the first thirteen amino acids of GGA3, which corresponds to exon-1 (Figure 1A) and cannot contribute to any inhibitory effect.

There was no evidence for either destabilization or upregulation of the remaining GGAs, as noted in a previous tissue-culture based study [28], and the level of adaptor protein 1 (AP-1) was similar between the different genotypes. Although it has been reported that depletion of any single GGA by RNAi causes a partial reduction in the levels of the accessory protein p56 in HeLa cells [31], we did not observe this effect in brain lysates isolated from the *Gga1*, *Gga3*, or *Gga1/3* null strains (Figure S2A). One possible explanation for these discrepancies is that the *Gga* null mice have adapted to the loss of individual GGAs differently than that observed in the tissue-culture based systems.

### Phenotypic characterization of *Gga1*, *Gga3*, and *Gga1/3* null mice

The *Gga1* and *Gga3* het mice were comparable to their wild-type (wt) counterparts at all ages. Mice homozygous null for *Gga1* or *Gga3* alleles exhibited reduced birthweights (Figure 1E), but no excess neonatal mortality (9% for *Gga1* and *Gga3* null mice vs 7% for wt). They gained weight somewhat slower than their wt littermates while their nose-rump lengths (NRL) were not different (Figure 1F and G). Their lifespans and fertility were normal.

By contrast, the *Gga1/3* null mice, while born at a normal ratio, had a 47% (17/36) mortality rate within 24 hours post-partum (Figure 1D). An additional 11% of these mice died within 3 weeks (4/36). The *Gga1* het/*Gga3* null mice also exhibited increased neonatal mortality (25/93) but this was half that observed with the *Gga1/3* null mice. The newborn *Gga1/3* null mice had a reduced birthweight similar to that of the *Gga1* and *Gga3* single null mice (Figure 1E). The surviving *Gga1/3* null mice gained weight even more slowly than the single nulls, and at 20 weeks exhibited a 38% reduction in weight compared to wt with no difference in NRL (Figure 1F and G). The *Gga1/3* null survivors had a normal lifespan but exhibited extremely poor fertility, which precluded generation of the *Gga1/3* null line.

We have previously reported that mice deficient in the gene (*Gnptab*) that encodes N-Acetylglucosamine-1-phosphotransferase, the enzyme that generates the mannose 6-phosphate recognition marker of lysosomal acid hydrolases, exhibit 6–40 fold elevations in their plasma levels of these hydrolases [33]. In order to assess if GGA deficiency also leads to similar enzyme hypersecretion *in vivo*, assays for five different lysosomal acid hydrolases were performed and the results are shown in Table 1. In contrast to the *Gnptab* null mice, the *Gga1*, *Gga3*, and *Gga1/3* null mice showed only a marginal elevation (1.6 to 2.2 fold) of a subset of the acid hydrolases. This indicates that lysosomal acid hydrolase sorting remains mostly intact even when two of the three GGAs are absent.

**Table 1:** Lysosomal enzyme activities in plasma of *Gga* null mice.

	wt	*Gga1* null		*Gga3* null		*Gga1/3* null	
Enzyme	Avg ± SD	Avg ± SD	Fold ↑	Avg ± SD	Fold ↑	Avg ± SD	Fold ↑
α-mannosidase	700±275	1138±315*	1.63	822±257	1.17	1165±377*	1.66
β-mannosidase	138±41	246±93*	1.78	227±79*	1.64	306±39*	2.22
α-galactosidase	26±14	28±8	1.08	25±9	0.96	48±9	1.85
β-galactosidase	15±5	26±11*	1.73	16±7	1.07	18±7	1.2
β-glucuronidase	22±8	23±6	1.05	19±4	0.86	18±4	0.82

Enzyme activity is expressed as nmole product formed/h/ml.

Fold ↑ = null activity/wt activity.

n = 28 for all mice except the *Gga1/3* null where n = 10.

* = p value<0.001.

### Generation of *Gga2* null mice

ES cell-line ID SYA176 containing a gene trap cassette inserted within intron-1 of the mouse *Gga2* gene was used to generate germline chimeras (Figure 2A). F1 het littermates in the C57BL/6J and 129/Ola mixed genetic background were crossed with the intent to generate *Gga2* homozygous null mice. Of the 88 offspring obtained on day 1 from the *Gga2* het matings, 28 expressed only wt message whereas 60 expressed both wt and chimeric message, as determined by RT-PCR (Figure 2B and E; Figure S1A). In addition, immunoblot analysis of brain lysates prepared from several litters of these day-1 offsprings failed to reveal any *Gga2* nulls (data not shown), in agreement with the RT-PCR data. The absence of mutant mice expressing only chimeric mRNA or lacking GGA2 protein indicated that inactivation of the *Gga2* gene causes embryonic lethality. To investigate the developmental stage at which the *Gga2* null fetuses die, embryos derived from timed *Gga2* het crosses were genotyped at different stages of gestation. The pregnant mice were euthanized at embryonic-day (E)9, E10, E11 and E12, followed by removal of the embryos and analysis of tissue lysates for the presence or absence of GGA2 by Western blotting. Of the 74 embryos analyzed, no *Gga2* null embryos were identified (Figure 2E). This indicates that the *Gga2* null embryos are dying before day E9 in this genetic background. An alternate explanation for our RT-PCR and Western blot data was that read-through transcription and splicing around the gene-trap cassette was occurring even in homozygous null mice resulting in wt mRNA and protein synthesis, as has been reported for a number of other gene-trap constructs [34], [35]. Since we were initially unable to genotype these *Gga2* mice at the genome level, the latter possibility could not be ruled out at the time. We were also concerned that perhaps this particular gene-trap cassette was causing embryonic lethality by a mechanism independent of the loss of GGA2, an inherent low-level risk of gene-trap mutagenesis [36].

For these reasons, we generated a second *Gga2* mutant strain using a different ES cell line. ES cell-line IST10483E10 containing a gene-trap cassette inserted within a known site in intron-1 of the mouse *Gga2* gene was used to generate germ-line chimeras in a C57BL/6J background (Figure 3A). In this background, genotyping of 169 newborn pups (Figure 3B) demonstrated that the mice were born in near accordance with the expected Mendelian ratio, although the homozygous group was moderately underrepresented (Figure 3C). This is likely due to the fact that 20 pups died and were cannibalized before they could be genotyped. To evaluate this possibility, an additional 99 newborns were genotyped within 12 hours of delivery to avoid loss of pups by cannibalism. In this set, the *Gga2* null mice represented 26% of the total (versus 29% wt and 45% het), confirming that the *Gga2* null mice in the C57BL/6NJ background were being born in the expected Mendelian ratio. Immunoblot analysis of brain lysates established the absence of GGA2 in the *Gga2* null newborn mice (Figure 3D), while the levels of the other GGAs, AP-1 and p56 were unchanged (Figure 3D; Figure S2B).

Strikingly, 73% of the mice that were homozygous for the gene-trap insertion died within the first 48 hours of birth and all of the remaining mice (with one exception) died within the first three weeks (Figure 3C). Dermal fibroblasts derived from the single survivor confirmed the null status of this mouse (Figure S3A). Thus, loss of GGA2 is lethal in both genetic backgrounds although it occurs during embryogenesis in one instance and during the early neonatal period in the other.

In order to evaluate the contribution of genetic background to the embryonic lethality of the SYA176 strain, *Gga2* het mice in the mixed background were backcrossed for 8 generations with wt C57BL/6J mice. Breeder pairs were set up with the eighth generation het mice and 45 offspring from these matings were sacrificed on day 1 and analyzed by a combination of genomic PCR and Western blotting (Figure 2F). Thus, determination of the insertion site of the gene-trap cassette within the SYA176 strain, accomplished while the backcrossing was being performed, allowed for accurate discrimination between wt, het and null genotypes in the backcrosses (Figure 2C and D). No genetic nulls were obtained by PCR (Figure 2D and F), or phenotypic nulls by immunoblot analysis (not shown), indicating that this *Gga2* strain remains embryonic lethal despite being backcrossed into the C57BL/6J background. One possible explanation for the discrepancy is that the TIGM *Gga2* mice are in the C57BL/6NJ background, which is not genetically identical to the C57BL/6J substrain (our SYA176 backcrossed mice), as described in the Jackson Laboratory Resource Manual [37]. In this regard, we have found no differences in brain expression of the three GGAs between C57BL/6J mice and C57BL/6J-129/Ola mixed genetic background mice (data not shown), suggesting that the strain or substrain differences we observe are not due to differential expression of the GGAs.

Since our *Gga1* null mice are also of mixed parentage, it was important to ascertain if genetic background could influence the phenotype of this strain. *Gga1* het mice that were backcrossed for eight generations into the C57BL/6J strain were intercrossed, and the results indicate that *Gga1* nulls in the black background are born in accordance with Mendelian ratio and are phenotypically normal (data not shown), demonstrating that the observed lethal phenotypes are unique to *Gga2*. A similar backcross into the C57BL/6J background is presently underway for the *Gga3* nulls.

### Phenotypic characterization of *Gga2* null mice

**Figure 4:**
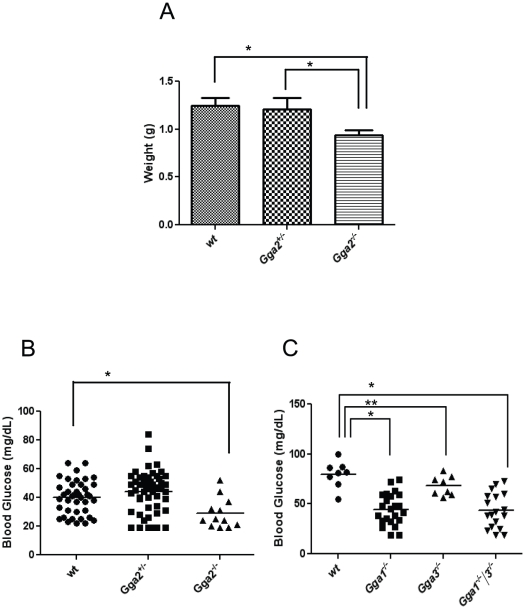
*Gga2* null mice exhibit low birthweights and hypoglycemia. A) Birthweights were obtained on day 1 and plotted according to genotype (wt, n = 42; *Gga2^+/−^*, n = 54, *Gga2* ^−/−^, n = 12). * represents p<0.001; as determined by Student's t-test. B) Blood glucose levels in TIGM *Gga2* newborn mice were determined on day 1 and plotted according to genotype (wt, n = 42; *Gga2^+/−^*, n = 54, *Gga2* ^−/−^, n = 12) * represents p<0.001; as determined by Student's t-test. C) Blood glucose levels in *Gga1* and *Gga3* newborn mice were determined on day 1 and plotted according to genotype (wt, n = 10; *Gga1* ^−/−^, n = 23; *Gga3* ^−/−^, n = 20; *Gga1* ^−/−^/*3* ^−/−^, n = 20). * represents p<0.001, ** represents p<0.05; as determined by Student's t-test.

Although 95% of the *Gga2* null mice derived from the TIGM cell line IST10483E10 died within 3 weeks of birth, histological examination revealed no obvious abnormalities. These *Gga2* null mice were significantly smaller than their littermates at birth (Figure 4A), but they were able to feed as evidenced by the presence of milk in their stomachs. Nevertheless, the null mice displayed striking hypoglycemia. Blood glucose levels obtained on postnatal day 1 averaged 28±10 mg/dL (n = 12) whereas the values of the wt and het mice were 41±12 mg/dL (n = 42) and 44±14 mg/dL (n = 54), respectively (Figure 4B). Interestingly, the *Gga1*, *Gga3* and *Gga1/3* null mice also exhibited hypoglycemia on postnatal day 1 (Figure 4C).

### Expression of GGAs

**Figure 5:**
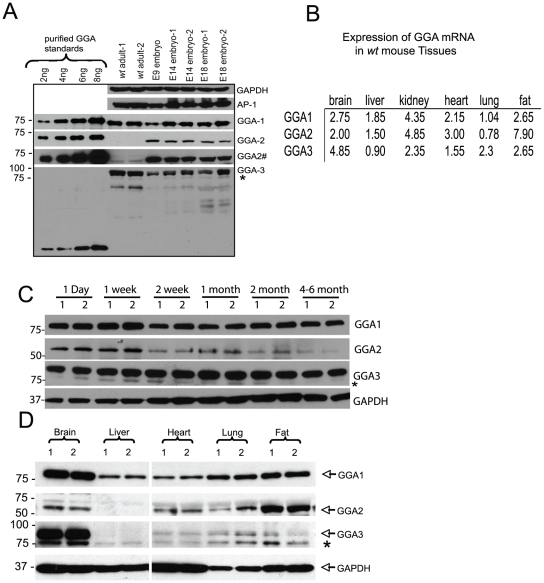
Tissue expression of GGA proteins. A) 25 µg of E9 whole embryo and E14, E18 and adult brain lysates along with 2–8 ng of purified standards were subjected to SDS-PAGE and immunoblot analysis. GGA1 and GGA2 purified standards were full-length Flag-tagged mouse proteins while the GGA3 standard was the mouse protein encoding only the VHS-GAT domain (see Figure S4A). Blots were probed for GGA1, GGA2, GGA3, AP-1 and GAPDH. Quantitation was derived from comparison to the GGA standards. # denotes a longer exposure of the GGA2 Western blot. B) The mRNA levels in mouse tissues were determined by quantitative realtime PCR and expressed relative to the expression of β-actin. First strand cDNA was synthesized from total RNA by reverse transcription as described in Materials & Methods. Realtime PCR was performed with SYBR Green Kit (ABI). The values presented are the average of two independent runs with total RNA isolated from the indicated organs of a wt mouse of C57BL/6J-129/Ola mixed genetic background. C) 25 µg of wt mouse brain lysates from mice (day 1 through adult stage) were subjected to SDS-PAGE and immunoblot analysis. Blots were probed as in panel A. D) 25 µg of wt adult mouse lysates (brain, liver, heart, lung and fat) were subjected to SDS-PAGE and immunoblot analysis. Blots were probed as in panel A.

One possible explanation for the embryonic/neonatal lethality associated with the *Gga2* null mice is that GGA2 is selectively expressed during embryogenesis and the neonatal period, resulting in the total loss of GGAs at these stages of development. Alternatively, GGA2 could be expressed at much higher levels than GGA1 and GGA3 during embryogenesis such that in its absence, the residual content of GGAs is insufficient to mediate some critical function. To address these possibilities, we developed a quantitative Western blot procedure to determine the level of each GGA during embryonic development and at adulthood. Brain extracts were prepared from wt embryos of mixed genetic background at various time points and analyzed by Western blotting to determine the content of the three GGAs at these stages, using purified GGAs 1–3 as standards. All three GGAs were detected at all stages of development (Figure 5A). GGA2 expression was highest at embryonic day 9 through the end of week 1, and then declined quite substantially achieving the lowest level in the adult brain (Figure 5A and C). By contrast, GGA1 and GGA3 expression increased from day E9 through day E18 and brain levels remained consistently high through adult stage. Importantly, when the expression level of the three GGAs was quantitated using the standards on each blot, it was apparent that the level of GGA2 was somewhat lower than the levels of GGA1 and GGA3 during the various stages of embryonic development (Figure 5A). Also, GGA2 was expressed at a much lower level than the other two GGAs in the adult rodent brain. A striking similarity was noted with adult human brain in that GGA2 was virtually undetectable whereas good expression was noted for GGA1 and GGA3 (Figure S3B). All three GGAs, on the other hand, were readily detected in human embryonic kidney 293 cells and Hela cells (Figure S3B). Mouse fibroblasts, in contrast, lack detectable GGA3, but express GGA1 and GGA2 (Figure S3A). Similarly, testis obtained from 2-week old wt mice had only trace amounts of GGA3 whereas the level of GGA1 was comparable to that found in the brain and GGA2 expression was higher than in the brain (data not shown). These results show that GGA expression can vary dramatically between tissues in both humans and mice. Also, the data are in agreement with microarray data showing that GGA2 mRNA expression is much higher in mouse embryos relative to adult brain (Mouse Gene Sorter, UCSC Genome Bioinformatics, http://genome.ucsc.edu/). Thus, at least in the brain, the level of GGA1 and GGA3 expression during embryogenesis would appear to be sufficient to compensate for the loss of GGA2 if the three GGAs were functionally redundant.

We also performed conventional real-time RT-PCR on total RNA prepared from wt adult tissues to determine the message levels of the three GGAs in brain, liver, kidney, heart, fat and lung. This analysis revealed that the GGA1 mRNA is most highly expressed in the kidney, brain and fat whereas GGA3 mRNA is expressed highest in brain followed by fat while GGA2 mRNA is most highly expressed in fat and the kidney (Figure 5B).

To further characterize the tissue specific expression of the GGAs in adult tissues, an immunoblot analysis was performed on tissues from wt mice. Consistent with the real-time RT PCR, GGA1 was most highly expressed in brain and fat whereas GGA3 had the highest expression in the brain and GGA2 in the fat (Figure 5D). The values obtained with the kidney extracts were quite variable which we attribute to protease activity, resulting in rapid degradation of the GGAs.

## Discussion

These data demonstrate that the GGAs are essential proteins in mammalian cells. They also provide two key findings concerning the issue of redundancy. First, while the individual loss of either GGA1 or GGA3 in mice is well-tolerated, the absence of GGA2 causes either embryonic or neonatal lethality, depending on the genetic background of the strain. And second, the combined loss of GGA1 and GGA3 results in a high incidence of mortality during the first day of life. The requirement for GGA2 indicates that this adaptor protein carries out a vital function that cannot be compensated for by GGA1 and GGA3. However, the nature of this critical function(s) is not clear at this time.

It is of interest that the expression level of GGA2 in the rodent brain is highest during embryonic development and early life but decreases substantially two weeks after birth. This is consistent with GGA2 serving a very important function during the embryonic and neonatal stage. This finding, however, does not obviate a role for GGA2 in adult animals since GGA2 is highly expressed in adipose tissue and fibroblasts (Figure 5B and D; Figure S3A). In contrast, brain expression of GGA1 and GGA3 remains consistently high from late embryogenesis through adulthood. The fact that the levels of GGA1 and GGA3 are somewhat greater in the brain than the level of GGA2 during development excludes the possibility that GGA2 is the sole GGA present in this organ. However, the GGA levels in the other tissues of the embryo could not be determined. In the C57BL/6NJ background, the *Gga2* null newborn mice are small, but histological examination of their organs on day 1 did not reveal any obvious abnormalities. These mice did have quite low blood glucose levels even though they had detectable milk in their stomachs indicating that they are able to suckle and take in milk. At this point the etiology of the low blood glucose is unknown and its possible contribution to the early demise of these mice is not clear.

Interestingly, the one female *Gga2* null mouse that survived is now an adult and has no obvious abnormalities to date other than being 25% smaller than its littermates. This indicates that GGA2 may be dispensable after the neonatal period and is consistent with the low expression level seen in rodent and human adult brains. This female mouse has been crossed with a *Gga2* het male and produced two small litters (5 and 3 pups, all of which died). The finding that the *Gga2* null mice in the mixed C57BL/6J and 129/Ola background (SYA176 strain) die before embryonic day 9 suggested that genetic background may influence the tolerance to the loss of GGA2. However, when the SYA176 mice were backcrossed into the C57BL/6J background, the embryonic lethal phenotype remained, as opposed to the neonatal lethality of the TIGM *Gga2* null mice. A closer look at the genetics of the two strains revealed that they are not identical as the SYA176 strain is in the C57BL/6J background while the TIGM strain is in the C57BL/6NJ background. It is thought that the many phenotypic differences among such closely related substrains could be due to as yet undiscovered genetic modifiers [37]. Furthermore, changes in gene expression clustered up to 28 MB of several knockout loci have been reported despite eleven generations of backcrosses into the C57BL/6 background, likely due to polymorphisms originating from the parental ES cells that alter gene expression [38]. Another remote possibility is that some important nucleotide element, such as a micro RNA, is situated in the 2 kb segment of intronic sequence between the upstream gene-trap insertion site in the SYA176 strain and the downstream insertion site in the TIGM strain, such that this critical element is only lacking in the chimeric GGA2 pre-mRNA in the SYA176 strain, and therefore contributes to its embryonic lethality [36].

The finding that loss of either GGA1 or GGA3 is well tolerated whereas the combined loss of both these GGAs results in a high incidence of neonatal lethality raises the possibility that these two GGAs share at least one vital function that cannot be carried out by GGA2. In this regard, both GGA1 and GGA3 are more similar to one another than to GGA2 in that both are highly phosphorylated, subject to autoinhibition and bind ubiquitin whereas GGA2 exhibits none of these properties [17], [23]–[25]. Moreover, crystallographic studies suggest that GGA1 and GGA3 VHS domains are more similar to each other than to the GGA2 VHS domain [39]. An alternative possibility for the partially lethal phenotype associated with the combined loss of GGA1 and GGA3 is that the expression level of GGA2 in some critical organ/tissue is not sufficient to overcome the loss of the other GGAs. It is of note that the *Gga1/3* null mice that lived beyond one month had a normal lifespan although their weight remained significantly lower than that of *wt* mice. Thus, as in the case of GGA2, GGA1 and GGA3 may not be required for survival after the neonatal-period.

While this manuscript was in preparation, two independent studies were published in which the single *GGA* gene in *Drosophila melanogaster* was knocked-down using GGA transgenic RNAi fly lines [40], [41]. Although the study by Hirst and Carmichael did not show any obvious phenotype when greater than 95% of the GGA is depleted in flies, the authors did note a gender specific higher expression of GGA in male flies [40]. In contrast, Eissenberg *et al* reported that GGA depletion resulted in lethality during early pupation under certain circumstances [41]. The basis for this discrepancy is not clear at this point.

The mammalian GGAs have been implicated in the trafficking of a number of transmembrane proteins including the mannose 6-phosphate receptors, sortilin, SorLA and BACE1 in *in vitro* studies. The knock-out mice described herein should be useful in determining the physiological roles of GGA1 and GGA3 in the intracellular pathways mediated by these receptors.

## Materials and Methods

All protocols involving the use of animals were in compliance with the National Institutes of Health's Guide for the Care and Use of Laboratory Animals and approved by the Animal Studies Committee in the Division of Comparative Medicine at Washington University School of Medicine in St. Louis (Protocol # 20100074). Mice were housed in a barrier facility maintained under standards meeting federal, state and local guidelines and under the supervision of licensed veterinarians.

### Generation of *Gga* null mice

The *Gga1* null mouse was generated from an ES clonal cell-line (cell-line ID AN0619) of 129P2/OlaHsd origin that was provided by the Sanger Institute (Cambridge, United Kingdom). This ES cell-line has a gene-trap cassette inserted within intron-7 of the mouse *Gga1* gene. The cell-line was expanded and injected into blastocysts derived from C57-BL/6J mice at the ES/Mouse Genetics Core facility of Washington University School of Medicine in St. Louis. Several male mice displaying at least 50% chimerism were bred with female C57-BL/6J mice for germ-line transmission. Founder *Gga1* het mice were initially identified by performing PCR across a segment of the neomycin gene within the gene-trap cassette. Since the precise location of the gene-trap within the 2.2 kb intron-7 of the *Gga1* gene was unknown, genomic DNA isolated from *Gga1* het mice was used to first accurately map this site by a combination of genomic PCR and DNA sequencing techniques (not shown). Upon acquiring this information, three primers were designed close to the insertion site, tested and verified, and subsequently used to genotype the progeny of inter-crosses between the *Gga1* het mice (Figure 1A and B; Figure S1A). Since these *Gga1* mice are in the C57BL/6J and 129/Ola mixed genetic background, they were backcrossed for eight generations with C57BL/6J wt mice to obtain an incipient congenic line.

Generation of the *Gga3* null mouse was initiated at the Mutant Mouse Regional Resource Center (MMRRC) in San Diego from an ES clonal cell-line (cell-line ID RRC067) of 129P2/OlaHsd origin that had a gene trap inserted in intron-1 of the mouse *Gga3* gene. Male chimeric mice were shipped to our institute for breeding with female C57-BL/6J mice. Founder *Gga3* het mice were initially screened by PCR across a segment of the neomycin gene. The insertion site of the gene trap within the 8.9 kb intron-1 of the *Gga3* gene was mapped precisely using genomic DNA isolated from a *Gga3* het mouse (not shown). PCR primers were then designed as before and used to genotype the progeny of a *Gga3* het inter-cross (Figure 1A and B; Figure S1A).

An ES clonal cell-line (cell-line ID SYA176) of 129P2/OlaHsd origin from the MMRRC that had a gene trap inserted in intron-1 of the mouse *Gga*2 gene was utilized to generate the *Gga2* het mice. Male chimeric mice were bred with female C57-BL/6J mice and *Gga2* founder pups were screened by PCR across a segment of the neomycin gene. We were initially unable to precisely map the insertion site of the gene trap cassette within the 8.8 kb intron-1 of the *Gga2* gene within this strain; consequently, we designed PCR primer sets that span exons-1 through 5, or exon-1 and the β-gal exon, and used these to genotype the progeny of *Gga*2 het inter-crosses using cDNA prepared by RT-PCR from either white blood cells or brain tissue (Figure 2A and B; Figure S1A). To verify the specificity of the PCR amplification generated with each primer set, PCR products were gel-isolated and sequenced (Figure S1B). When the position of the gene-trap cassette was eventually determined, PCR primers were designed to span the insertion site allowing us to clearly distinguish between *Gga2* wt, het and null mice (Figure 2C and D; Figure S1A). Since these *Gga2* het mice, like the *Gga1* strain, are also of C57BL/6J and 129/Ola mixed genetic background, they were backcrossed for eight generations with C57BL/6J wt mice to obtain an incipient congenic line.

A second ES clonal cell-line (cell-line ID IST10483E10) of C57BL/6N origin from Texas A&M Institute for Genomic Medicine (TIGM, College Station, TX) that had a gene trap inserted in intron-1 of the mouse *Gga*2 gene was utilized to generate the *Gga2* (TIGM) het mice. Male chimeric mice in the black background were bred with female C57-BL/6J mice and *Gga2* founder pups were screened by PCR across a segment of the neomycin gene for the *Gga*2 het genotype. The position of the gene-trap cassette within intron-1 of the *Gga2* gene in this strain was available from TIGM, which facilitated the design of primers to enable genotyping of the progeny of a *Gga2* het inter-cross (Figure 3A and B; Figure S1A). The sizes of all PCR and RT-PCR fragments associated with the various *Gga* null mice in this study are presented in Figure S2A.

### PCR conditions for genotyping of mice

For genotyping of mice, genomic DNA was prepared from toe/tail tissue by boiling in 300 µl of alkali (1∶200 diluted solution of 10N NaOH) for 1 hr, followed by neutralization with 25 µl of 1 M Tris.Cl, pH 8.0. 1 µl of this crude DNA extract was used for PCR analysis using the various primer sets shown in Figure S1A. PCR cycling conditions were as follows: step 1–95°C 2.5 min; step 2–95°C 50 sec; step 3–62°C 50 sec; step 4–72°C 1 min; step 5 - repeat cycles two through four 32 times; step 6–72°C 10 min. All PCR products were analyzed on 2% agarose gels. The fragment sizes of the PCR products generated with the different primer sets are shown in the supplementary data.

### Harvesting mouse tissues and Western blot analyses

Adult mice were euthanized by CO_2_ inhalation while newborns and embryos were sacrificed by decapitation. For the embryo analysis, day E9 whole embryo, E12 and E14 whole head and E18 brain tissue were used. Adult brain, kidney, liver, heart, white fat and lungs were removed and weighed. 100 mg of each tissue sample was rinsed in phosphate-buffered saline (PBS) and five volumes of PBS with 1% Triton X-100 (PBS-T) containing proteinase inhibitors (Roche) were added. Tissues were homogenized in 1.7 ml microcentrifuge tubes on ice using a Teflon pestle. Homogenates were centrifuged at 15,000× *g* for 15 min and the supernatants were removed, and the protein concentrations determined. All samples were diluted to 5 mg/ml before being boiled in SDS sample buffer for gel loading. Proteins were resolved by SDS-PAGE, transferred to nitrocellulose membrane and probed with the following antibodies: anti-GGA1 (GGA1 H-215 for both mouse and human proteins) (Santa Cruz), anti-GGA2 (GGA2 H-175 for mouse protein) (Santa Cruz), anti-GGA2 (cat #612613 for human protein) (BD Transduction Laboratories), anti-GGA3 (made against purified VHS-GAT domain of recombinant mouse GGA3 to detect mouse protein; see Figure S3A and B), anti-GGA3 (cat#612311 for human protein) (BD Transduction Laboratories), anti-AP-1 (cat#610385 for mouse and human proteins) (BD Transduction Laboratories), anti-p56 (custom synthesized for us by Covance; see Supplementary data), β-tubulin (Sigma) and GAPDH (Sigma).

### Analyses of GGA mRNA in mouse tissues

Tissues were homogenized in TRIzol reagent (Invitrogen) and total RNA was extracted according to the manufacturer's instructions. Superscript First Strand Synthesis system (Invitrogen) was used to synthesize cDNA. The expression of mRNA was examined by quantitative PCR analysis using a 7500 Fast Real-Time PCR machine (Applied Biosystems). Amplimers for GGA1, GGA2 and GGA3 (ABI; catalog # PPM35135A, PPM27582A, PPM30931A, respectively) were employed using SYBR Green detection. The relative mRNA expression was normalized by measurement of the amount of β-actin mRNA in each sample. β-actin primer 5′- GACCCTGAAGTACCCCATTGAAC AND 5′-CACGCAGCTCATTGTAGAAGGT. The PCR cycling conditions were as follows: step 1–95°C 5 min; step 2–94°C 30 sec; step 3–60°C 30 sec; step 4–72°C 20 sec; step 5–78°C 1 sec for plate reading; step 6 – repeat cycles two through five 40 times; step 6–72°C 10 min.

### Lysosomal enzyme assays

Lysosomal hydrolase activities were measured by fluorometric analysis as described previously [33]. Briefly, various 4-methylumbelliferyl-coupled substrates (5 mM) were incubated with plasma samples isolated from the indicated mice in a 50 mM citrate buffer containing 0.5% TritonX-100, pH 4.5, at 37°C. Reactions were terminated by addition of 0.1 M glycine-NaOH solution, pH 10.3, and the resulting fluorescence was read at 495 nm. Activities are expressed as nanomoles of methylumbelliferone released per hour per milliliter of plasma.

### Glucose levels

Glucose levels were determined with the One Touch Glucose monitoring kit (Johnson & Johnson) using 10 µL of blood isolated from tails of newborn (day 1) mice. Blood glucose levels are represented as individual points with an average ± standard deviation.

### Histology

Paraformaldehyde-fixed newborn mice or tissues isolated from three week- old mice were cut in 5 µm sections. Sections were applied to slides and stained with Hematoxylin–eosin.

### Expression and purification of mouse GGAs

Full-length mouse GGA1 and GGA2 and the VHS/GAT domains of mouse GGA3 with a Flag epitope were expressed in SF9 insect cells and purified to homogeneity on anti-Flag M2 affinity gel according to the manufacturers protocol (Sigma). Protein concentrations were determined by the Bradford assay (BioRad) and the purified proteins were used as standards in the immunoblot analysis.

## Supporting Information

Figure S1 ***Gga*** **and gene-trap primers used to genotype mice.** **A.** The *Gga1*, *Gga3*, *Gga1/3*, *Gga2* (SYA176) and *Gga2* (TIGM-IST10483E10) genotypes were assessed by PCR analysis of genomic DNA as described under Materials and Methods. Additionally, *Gga2* (SYA176) was typed by RT-PCR of cDNA prepared from blood or brain tissue. Two sets of primers detected the wt or *Gga1^−^* ^/*−*^ allele. *Gga1* primers fwd1 and rev1 yielded a 444-bp product specific for the wt allele, and primers fwd1 and rev2 yielded a 520-bp fragment specific for the *Gga1* allele containing the gene-trap. Two sets of primers detected the wt or *Gga2^−^* ^/*−*^ allele. For the SYA176 strain, *Gga2* primers fwd1 and rev1 yielded a 348-bp product specific for the wt allele, while primers fwd1 and rev2 yielded a 592-bp fragment specific for the *Gga2* allele containing the gene-trap. For the TIGM strain, *Gga2* primers fwd1 and rev1 yielded a 615-bp product specific for the wt allele, while primers fwd1a and rev2 yielded a 326-bp fragment specific for the *Gga2* allele containing the gene-trap. Two sets of primers detected the wt or *Gga3^−^* ^/*−*^ allele. *Gga3* primers fwd1 and rev1 yielded a 494-bp product specific for the wt allele, and primers fwd1 and rev2 yielded a 465-bp fragment specific for the *Gga3* allele containing the gene-trap. For RT-PCR, two sets of primers detected the wt or chimeric mRNA. *Gga2* primers fwd1 and rev1 yielded a 464-bp product specific for the message transcribed from the wt allele, while primers fwd1 and rev2 yielded a 555-bp fragment specific for the message transcribed from the *Gga2* allele containing the gene-trap. **B.** To verify the specificity of the PCR amplification products generated from the cDNA of the SYA176 strain, PCR products were gel-isolated and sequenced. B1 corresponds to wt *Gga2* mRNA, while B2 corresponds to *Gga2* exon-1 spliced to the *β-gal* exon.
(EPS)

Figure S2 **Analysis of p56 in** ***Gga*** **null mice.** **A.** Analysis of p56 in *Gga1*, *Gga3* and *Gga1/3* brains of adult mice. 25 µg of total protein from whole brain lysates was resolved by SDS-PAGE, transferred to nitrocellulose and probed with anti-p56 rabbit polyclonal antibody made against the synthetic peptide described by Mardones et al. [31] and custom-synthesized for us by Covance. GAPDH was detected with an anti-GAPDH mouse monoclonal antibody. **B.** Analysis of p56 in *Gga2* (TIGM) brains of day 1 pups. Samples were prepared and analyzed as in A.
(EPS)

Figure S3 **Low expression of GGA2 in mouse and human adult brain.** **A.** Comparison of GGA2 expression between mouse adult brain and primary dermal fibroblasts shows greatly diminished level in brain. In contrast, GGA1 is expressed well in both tissues while GGA3 shows high expression in brain but extremely low level in fibroblasts. The absence of the GGA2 signal in fibroblasts derived from the single surviving *Gga2* null mouse demonstrates the specificity of the signal. **B.** Similar to mouse, the level of GGA2 expression is below the detection limit in human adult brain. However, it is also quite low in fibroblasts, but readily detected in HEK 293 and Hela cells. In contrast, GGA1 and GGA3 are expressed at good levels in all four tissues/cell-types examined.
(EPS)

Figure S4 **Production and testing of mouse-specific GGA3 pAb.** **A.** Since none of the commercial anti-GGA3 antibodies specifically detected mouse GGA3 in western blot applications, mouse GGA3 (residues 1–345) was expressed in Sf9 insect cells, purified to homogeneity and used to immunize either *Gga*3^−/−^ or wt (control) mice for antibody production. This strategy elicited an antibody response in some of the *Gga3* ^−/−^ mice but none of the control animals. Western blot analysis of mouse brain lysates using the mouse polyclonal anti-sera showed that GGA3 expression was reduced in the *Gga*3^+/−^ strain and ablated in the *Gga*3^−/−^. There was also an additional band that migrated slightly above the 75 Kd marker that proved to represent cross-reactivity with GGA1, as shown in panel B. **B.** The lower band proved to be a GGA1 cross-reacting band, since it was absent in *Gga1* ^−/−^ and reduced in *Gga1* ^+/−^ brain extracts.
(EPS)
